# A Teach-Discover-Treat Application of ZincPharmer: An Online Interactive Pharmacophore Modeling and Virtual Screening Tool

**DOI:** 10.1371/journal.pone.0134697

**Published:** 2015-08-10

**Authors:** David Ryan Koes, Nicolas A. Pabon, Xiaoyi Deng, Margaret A. Phillips, Carlos J. Camacho

**Affiliations:** 1 Department of Computational and Systems Biology, University of Pittsburgh, Pittsburgh, PA, United States of America; 2 Department of Pharmacology, University of Texas Southwestern Medical Center at Dallas, 6001 Forest Park Blvd, Dallas, TX, United States of America; University of South Alabama Mitchell Cancer Institute, UNITED STATES

## Abstract

The 2012 Teach-Discover-Treat (TDT) community-wide experiment provided a unique opportunity to test prospective virtual screening protocols targeting the anti-malarial target dihydroorotate dehydrogenase (DHODH). Facilitated by ZincPharmer, an open access *online interactive pharmacophore search of the ZINC* database, the experience resulted in the development of a novel classification scheme that successfully predicted the bound structure of a non-triazolopyrimidine inhibitor, as well as an overall hit rate of 27% of tested active compounds from multiple novel chemical scaffolds. The general approach entailed exhaustively building and screening sparse pharmacophore models comprising of a minimum of three features for each bound ligand in all available DHODH co-crystals and iteratively adding features that increased the number of known binders returned by the query. Collectively, the TDT experiment provided a unique opportunity to teach computational methods of drug discovery, develop innovative methodologies and prospectively discover new compounds active against DHODH.

## Introduction

The Teach-Discover-Treat (TDT) competition was created to encourage the development of high-quality computational chemistry tutorials within the context of drug discovery for neglected diseases. Here we present our winning interactive pharmacophore modeling virtual screening workflow for targeting the anti-malaria dihydroorotate dehydrogenase (DHODH) enzyme and report the results of the follow-on experimental validation from the 2012 TDT competition.

Unlike mammalian cells, which have salvage enzymes, the malarial parasite *Plasmodium falciparum* depends on *de novo* synthesis of pyrimidines [1]. DHODH catalyzes the rate-limiting fourth step of pyrimidine synthesis and inhibitors of this enzyme are effective against both normal and drug-resistant strains of the parasite in mouse models [2, 3]. The challenge addressed in the TDT competition was to use existing structures of DHODH [3, 4] to identify commercially available inhibitors with chemical scaffolds distinct from existing inhibitors [2, 5–7]. In addition to the publically available DHODH structures, the TDT challenge provided a congeneric series of 192 triazolopyrimidine DHODH inhibitors with activities that spanned four orders of magnitude. Besides the virtual screening component, the TDT competition included a binding pose prediction exercise for a non-triazolopyrimidine inhibitor [8], N-(3,5-dichlorophenyl)-2-methyl-3-nitrobenzamide, referred to by the TDT organizers as compound 6, that, at the time of the exercise, had no published structure.

To address the dual education and drug discovery goals of TDT, we leverage the user friendly and interactive capabilities of our server ZincPharmer [9] to introduce students to the problem of virtual screening and computational drug discovery. More specifically, ZincPharmer supports the design of pharmacophore models for a given protein ligand interaction structure. A pharmacophore describes the spatial arrangement of the essential features of a biological interaction, such as the hydrophobic, hydrogen bond, charged, or aromatic features. Thus, in the present challenge, the students created pharmacophore models by identifying the most relevant features from co-crystals of the DHODH enzyme with known small molecule inhibitors and tested their models against a benchmark compound database.

**Fig 1:**
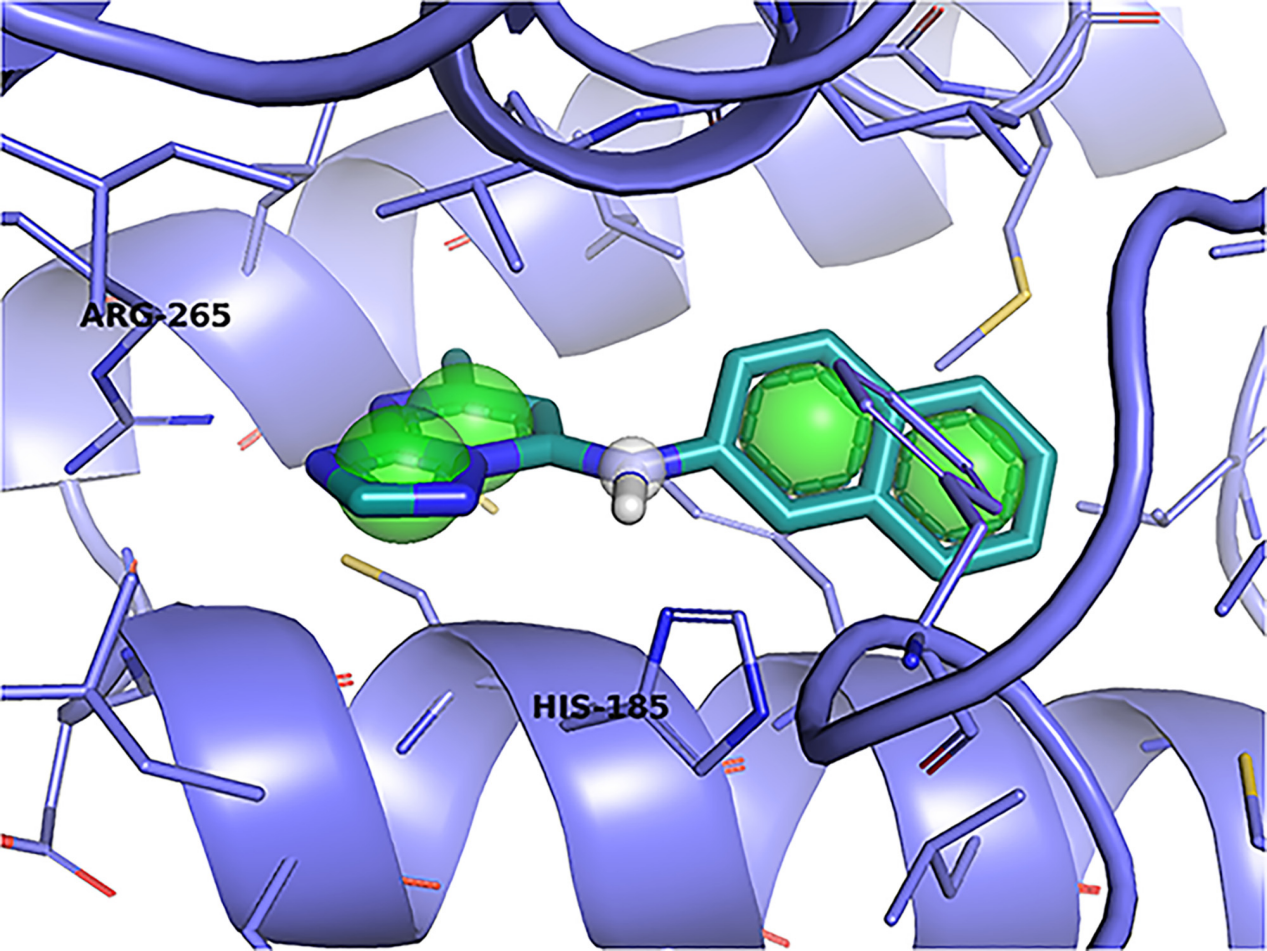
A pharmacophore derived by a student from a structure of DHODH bound to an inhibitor (PDB 3I65). The pharmacophore consists of hydrophobic features (green spheres) and a hydrogen donor feature (white sphere). This pharmacophore was used as part of a virtual screen for novel inhibitors.

The goal of the teaching unit we developed is to introduce students to computational drug discovery while teaching basic fundamentals of molecular interactions. Students are actively engaged in the material through a competitive, interactive pharmacophore modeling exercise targeted at the DHODH enzyme. The best identified pharmacophore, shown in Fig 1, was the result of a novel approach that entailed exhaustively building and screening sparse pharmacophore models comprising of a minimum of three features for each bound ligand in all available DHODH co-crystals and iteratively adding features that increased the number of known binders returned by the query. This design was then used to screen a large collection of commercially available compounds and to predict the bound structure of a non-triazolopyrimidine inhibitor. The matching compounds were then energy minimized and ranked with respect to DHODH using two distinct scoring functions. Two ranked sets of the top 1,000 compounds identified by each scoring function were submitted as part of our entry in the TDT submission. As one of the winners of the TDT competition, a subset of 167 of our virtual screening hits was selected for experimental validation. Among the screened compounds, 27% demonstrated inhibition of at least 10μM and several have novel chemical scaffolds. Moreover, the prospective prediction of the bound structure of a novel non-triazolopyrimidine inhibitor resulted in a model with a heavy atom RMSD of 1.2 Å compared to the crystal structure.

The TDT experiment provided the first independent validation of ZincPharmer, the only open access interactive technology capable of screening the ZINC database [10] in tens of seconds. Our user-friendly platform allowed for a crowd-sourcing [11] experiment that successfully potentiated iterative and exact pharmacophore matching [12], model modification and fast database query for the given target. These properties, which are not readily available with other technologies, ultimately led to the implementation of an innovative and easily applicable methodology. Alternative approaches typically emphasize discretization, scoring, statistical methods, and fingerprints that are often challenging to apply for non-expert [13, 14]. Ultimately, very few screening technologies are independently validated to the scale afforded by TDT, and the successful predictions prospectively confirmed here speak to the usefulness of the technology. Equally important, several new hits to this target with unmet clinical needs were uncovered from historical libraries, and could hopefully spear efforts to new therapies.

## Materials and Methods

### Teaching Unit

The teaching unit can be administered in a single 90–120 minute session and consists of a didactic lecture and a hands-on component. Only a basic, high-school level of scientific understanding is required from the students. Since the unit is self-contained, it is particularly well-suited for scientific outreach or other venues where in-depth follow-up (e.g., homework) is not an option. Students are taught the basic physical and biological properties of the most common pharmacophore features (hydrophobics, hydrogen bonds, charges, aromatics). As much as possible, the effects of these microscopic interactions are connected to commonplace macroscopic observations. The PowerPoint slides for this lecture are available at http://zincpharmer.csb.pitt.edu/tdt/TDT_computational_drug_discovery_lecture.pptx and are licensed under the Creative Commons Attribution 3.0 License.

### Interactive and Competitive Pharmacophore Modeling

After the molecular interaction lecture, students are tasked with finding an informative pharmacophore model for DHODH. Pharmacophore models are developed using a structure-based approach where students examine the interactions of DHODH inhibitors of known structure, choose what they think are the most significant features of the interaction, and then use this pharmacophore model to screen a benchmark library of inactive and active compounds. The quality of the model is assessed, and students post their results in real-time to an online leaderboard as part of a class-wide competition that serves to further engage the students in the activity.

### Benchmark Library

In order to evaluate the quality of the models developed by students, we created a database of inactive and active compounds. We used both the inactive and active compounds of PubChem BioAssay 1175, which targeted the inhibition of DHODH in *Plasmodium falciparum*, as well as the 193 active compounds provided by the TDT organizers. This resulted in a set of 5495 compounds, of which 276 were active. Three-dimensional conformers were generated from this set using OpenEye's omega program (version 2.4.6) with the options-maxconfs 25-rms 0.7-strict false resulting in 113,648 conformers. Pharmer [15] was then used to build a pharmacophore database of these conformers to support rapid searching (less than a second for most queries).

**Fig 2:**
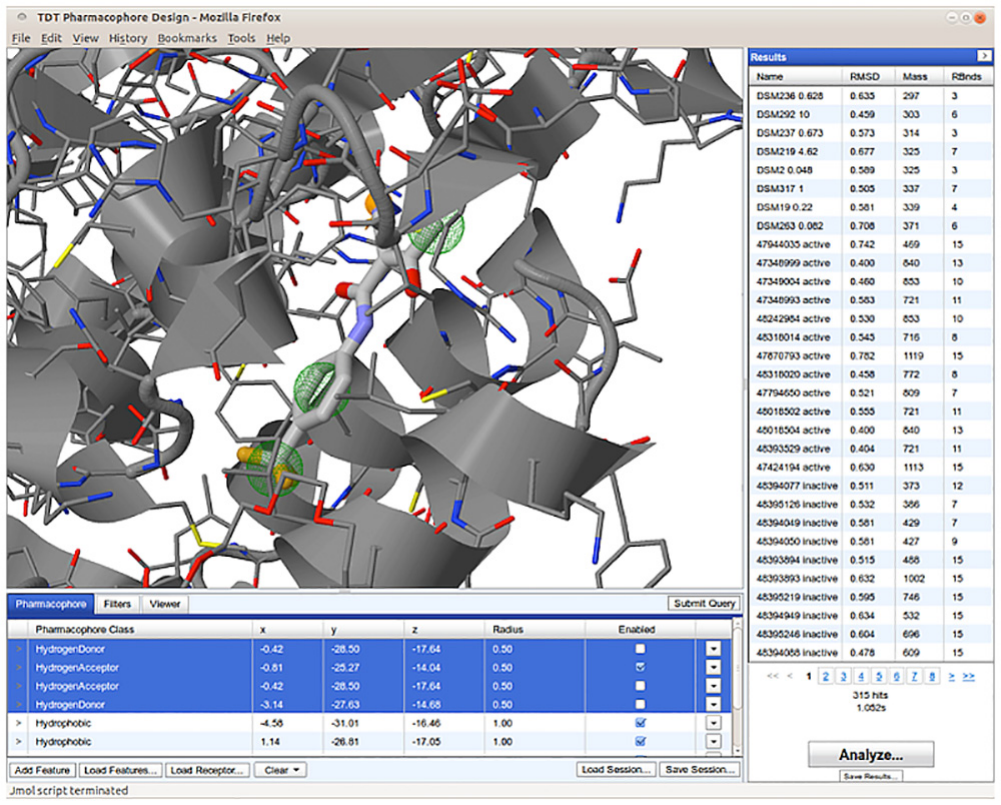
The ZINCPharmer based interactive pharmacophore modelling interface used by the students to competitively develop an informative pharmacophore model.

Students used a modified version of the ZINCPharmer [9] online pharmacophore visualization and editing platform, shown in Fig 2, to interactively search this benchmark library using any modern web browser. The quality of a pharmacophore model was assessed by computing its recall, precision, specificity, accuracy, enrichment factor, and F1 score with respect to the benchmark library. The F1 score, the geometric mean of the precision and recall, was used to rank the quality of the pharmacophore models. Students competed in real-time to produce a pharmacophore that maximized the F1 score while still retrieving compound 6. Retrieval of compound 6 was required since part of the TDT competition was predicting its pose. Additionally, this requirement had the side-effect of eliminating pharmacophores that are overly specific to triazolopyrimidine scaffolds.

### Structure-Based Modeling

As a basis for structure-based pharmacophore modeling, we selected six PDB structures of bound DHODH (1TV5, 3I65, 3I68, 3I6R, 3O8A, 3SFK). Students then worked to identify the most relevant pharmacophore features present in each complex. The general approach used by the winning student was to construct a sparse pharmacophore model that returned all or most known DHODH-active compounds, including compound 6, and then to iteratively refine the model to improve the enrichment of actives vs. non-actives in the query results, as measured by the F1 score. Sparse pharmacophore models, comprised of three features, were built for each bound ligand in all available DHODH co-crystals. Limiting the number of pharmacophore features in these initial models reduced the number of possible models and allowed more rapid sampling of the search space via a simulated annealing-like procedure. This facilitated the discovery of optimal sparse queries for each co-crystal that returned the maximal number of known binders including compound 6. At this stage in the process, query results were evaluated only in terms of the presence of compound 6 and the total number of active compounds returned; inactive compounds in the query results were ignored.

In what follows we describe in detail the procedure that led to the design of the optimal model. Inhibitor-bound DHODH co-crystal PDB: 3I65 resulted in the best pharmacophore models, while the other co-crystals failed to consistently retrieve compound 6 among the hits.

**Fig 3:**
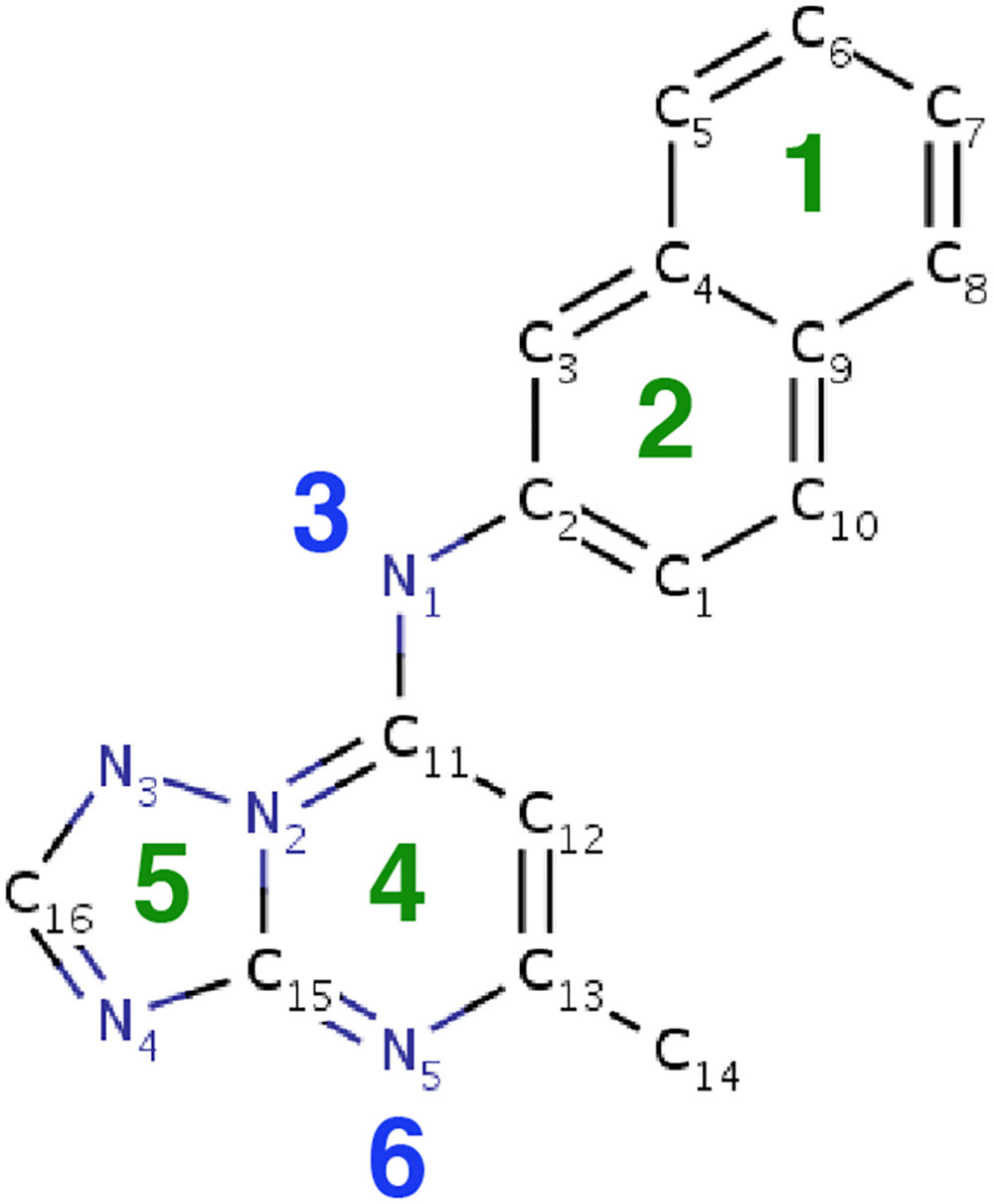
Chemical structure of DHODH inhibitor 5-methyl-7-(naphthalene-2-yl-amino)-1H-[1,2,4]triazolo[1,5-a]pyrimidine-3,8-diium. The six pharmacophore features used to define the sparse model search space are labeled. Green labels refer to hydrophobic groups, blue labels refer to hydrogen bond features.

The initial model space considered for PDB: 3I65 consisted of six pharmacophores, which were rationally chosen based on the inhibitor chemistry and the chemical environment of the binding site. This limited the search space to a total of 20 sparse models–each containing three pharmacophores, which could be rapidly evaluated and ranked by recall and the presence of compound 6. Fig 3 depicts the structure of the DHODH inhibitor on which the pharmacophore model was based.

The six pharmacophores used to define our sparse model search space are labeled in Fig 3, and are described below. Of the initial six pharmacophores considered, two comprised the polar contacts made between DHODH and the small molecule inhibitor: (3) a hydrogen donor at the central N1 atom, which forms a hydrogen bond (HB) with HIS185, and (6) a hydrogen acceptor at the terminal N5 atom, which forms a HB with ARG265. Two additional pharmacophores (1,2) are hydrophobic groups on the inhibitor’s naphthalene rings, chosen because they fit favorably in the hydrophobic cavity made by DHODH residues PHE188, LEU189, LEU197, PHE1127, ILE237, LEU240, LEU531, and MET536 (see Fig 2). The final two pharmacophores (4,5) are hydrophobic groups on the nitrogen rings of the inhibitor, chosen because they sit favorably in the hydrophobic cavity made by LEU172, LEU176, CYS184, ILE272, and VAL532.

The twenty possible sparse models were used to screen the virtual library and were then ranked by recall–the fraction of true binders returned in search results. Models that did not return compound 6 were discarded. The top five sparse models are listed in Table 1. It was noted that 7/10 sparse models containing the hydrogen acceptor feature number 6 did not return compound 6 in the query results, suggesting that the structure of compound 6 was generally incompatible with pharmacophore models containing this feature.

**Table 1:** Query results for the top five sparse pharmacophore models.

Model Features	Recall	F1 Score	Compound 6
1,2,4	89.49	.124	yes
1,2,5	86.59	.117	yes
1,5,6	90.94	.102	yes
2,4,5	89.86	.116	yes
2,5,6	98.91	.102	yes

For each of these top five sparse models, we then added the remaining pharmacophore features one by one to identify the optimal 4-feature models. These models were evaluated with the F1 score and the presence of compound 6. The process was then repeated with top two 4-feature models in order to find the optimal 5-feature model. The top 4- and 5-feature models are listed in Table 2. This approach resulted in the model shown in Fig 1, consisting of pharmacophores 1-to-5.

**Table 2:** Query results for top four- and five-feature pharmacophore models.

Model Features	Recall	F1 Score	Compound 6
2,3,4,5	60.51	.592	yes
1,2,3,4,5	47.46	.642	yes

### Virtual Screening

Pharmacophore models can quickly filter a large library of compounds, but are less useful for rank ordering the results and refining the pose. The virtual screen requested by the TDT challenge required 1,000 rank ordered compounds selected from a provided eMolecules compound set. To perform this virtual screen, we generated conformers using OpenEye omega version 2.4.3 and the command-line options-maxconfs 10-rms.7. These conformers were then incorporated into a Pharmer database and screened using the pharmacophore shown in Fig 1 resulting in 44,809 unique hits. The returned poses were then minimized using smina [16], a fork of AutoDock Vina [17]. We filtered out any compound poses where the RMSD between the pharmacophore aligned pose and the minimized pose was greater than 1Å so that the results remained faithful to the pharmacophore model.

We generated two sets of rank ordered compounds. The first set consisted of the top thousand compounds as determined by the default AutoDock Vina scoring function [17], which was parameterized to maximize docking pose prediction performance and contains terms for steric complementarity, undirected hydrogen bonds, hydrophobic interactions, and the number of rotatable bonds. The second set consisted of the top thousand compounds as determined by a custom scoring function[16] that was parameterized for affinity prediction against the 2010 CSAR-NRC HiQ dataset[18] and includes terms for desolvation, van der Waals, undirected hydrogen bonds, and the number of rotatable bonds. All compounds were energy minimized with respect to the 3I65 receptor structure that the pharmacophore model was based on.

### Pose Prediction

**Fig 4:**
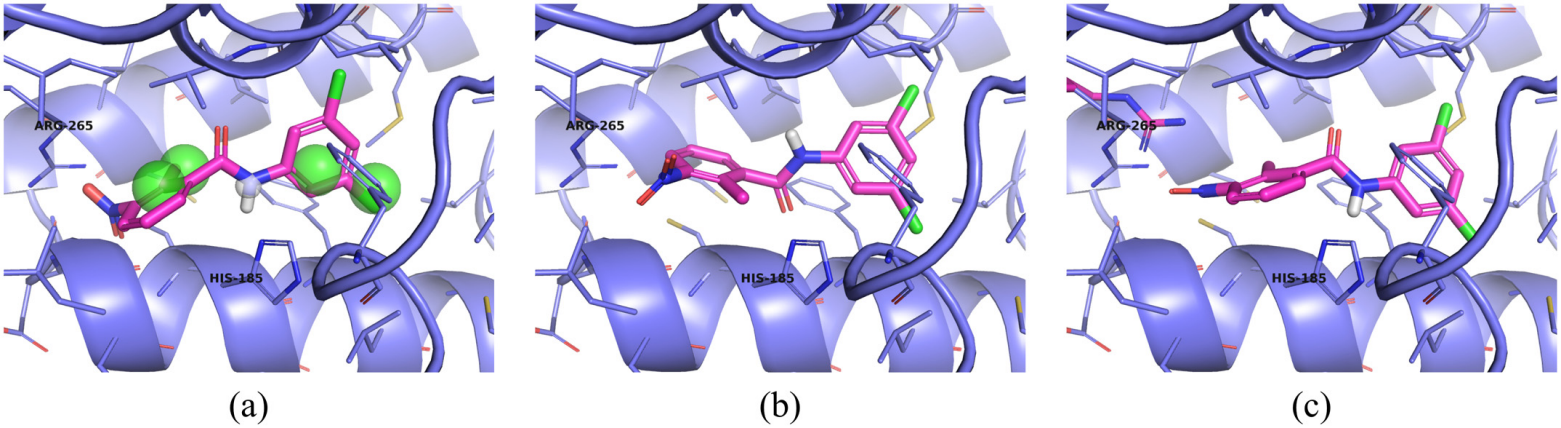
Pose prediction of compound 6. Receptor structure and binding site residues of 3I65 are shown in blue. Compound 6 is shown in magenta sticks. (a) Compound 6 aligned to the pharmacophore of Fig 1. The compound makes a hydrogen bond with HIS-185. (b) After minimization, the pose has twisted so that the hydrogen bond to HIS-185 is broken. (c) When the pharmacophore aligned posed is minimized with a flexible ARG-265, which sterically clashes with the initial pose, a less dramatic movement is observed and the hydrogen bond to HIS-185 is maintained.

In order to predict the pose of compound **6**, we minimized the pharmacophore aligned pose of this compound, shown in Fig 4(a), using smina [16] with the AutoDock Vina scoring function [17]. However, as shown in Fig 4(b), the resulting minimized pose deviated from the pharmacophore-aligned pose by more than 2.5Å RMSD, as computed by the obrms tool of the OpenBabel toolkit [19]. In particular, the minimized pose moved the requested hydrogen donor away from the accepting histidine (HIS-185). Since these movements seemed to be motivated by a clash with ARG 265, we minimized with the side chain of this residue set as flexible. The result is shown in Fig 4(c). Although this pose deviates from the pharmacophore-aligned pose by 2.0 Å RMSD, it maintains the correct orientation to make a hydrogen bond with HIS-185.

### Experimental Validation

After the conclusion of the TDT competition, compounds were selected from the winning entries for testing.

### Enzyme kinetic analysis

Protein PfDHODHΔ384–413 (amino acids 162–565) was expressed and purified from E. coli BL21 phage-resistant cells (Novagen) using plasmid (pET28b-TEV- PfDHODΔ384–413) as described previously [20]. Compounds that resulted from the TDT competition were ordered from eMolecules and were dissolved to 10 mM using methanol. An end-point assay monitoring the reduction of 2,6-dichloroindophenol (DCIP) was used for the 384-well plates high-throughput screening. The DCIP dye-based assay was performed as previously described [21] using the following assay buffer (100 mM HEPES pH 8.0, 150 mM NaCl, 10% glycerol, and 0.1% Triton) and substrates (0.2 mM dihydroorotate (DHO), 0.02 mM CoQ_D_, and 0.12 mM DCIP). Reduction was followed at 600 nm (ε = 18.8 mM^–1^ cm^–1^). To screen for compound activity, 40μl of assay buffer and 1μl of compound (2 final different concentrations were screened, 10 and 1 μM) were transferred into 384-wells plate using a Biomek FX robotic liquid-handling device (Beckman Instruments), then 10 μl of enzyme solution (20 nM final enzyme concentration in assay buffer) was transferred to the plate. The reaction was incubated for 10 min at room temperature and then stopped by the addition of 10 μl 10% SDS. The absorbance of each well was measured at OD595 nm using a microplate reader (Bio-Tek Instruments), and the data were exported to an Excel spreadsheet for analysis. No-drug Me2SO and 0.33 μM DSM1 were used in the experiment as controls. A hit was recorded when compound (10 μM) reached 40% of DSM1 inhibition of enzymatic activity.

## Results

### Pedagogical Evaluation

We presented our teaching unit to a class of undergraduate researchers and a class of high school researchers who were involved in summer programs at the University of Pittsburgh. Students remained engaged with the pharmacophore modeling exercise for the full duration of the class. Overall, the class was well-received and ranked highly in student evaluations (third out of eleven lectures). In fact, one student noted: “This was my favorite class presented in the series.” The majority of high school students could correctly identify the definition of a pharmacophore at the conclusion of the program several weeks later.

### Comparison of the prospective structure prediction and the subsequently resolved crystal structure

**Fig 5:**
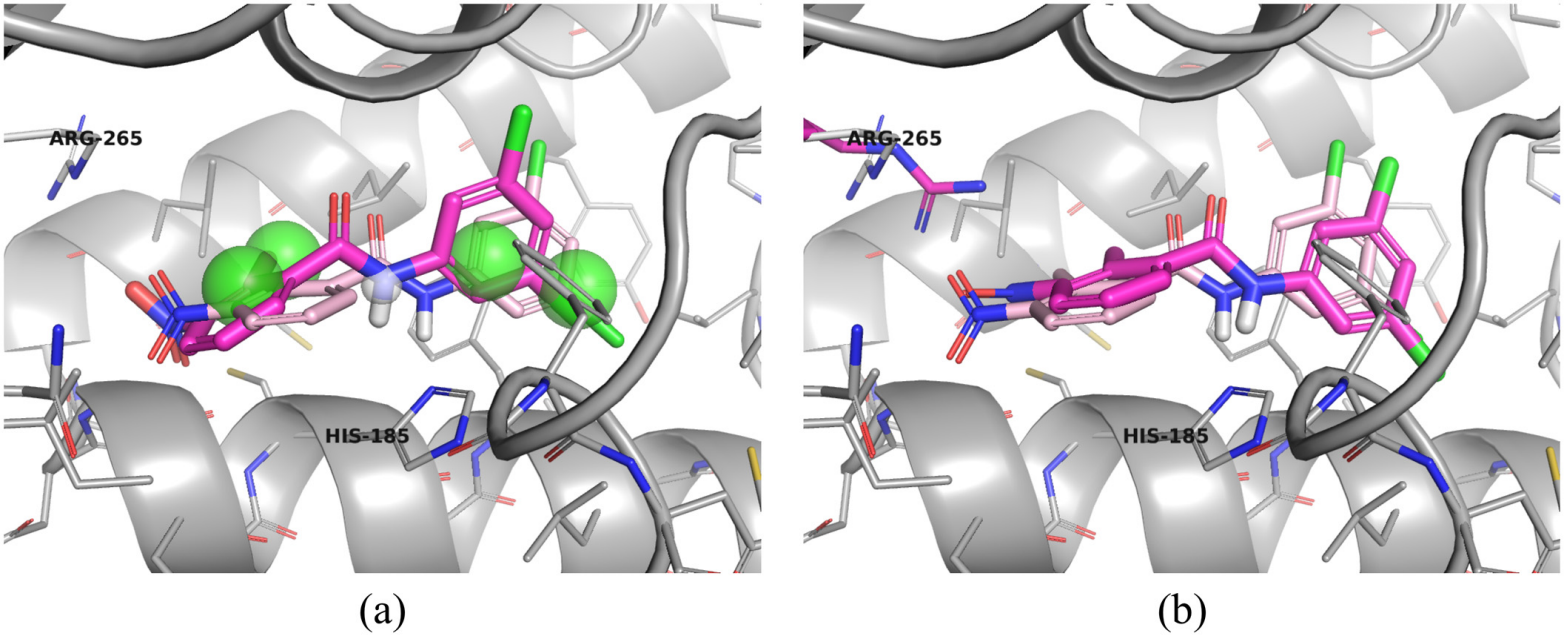
Pose prediction results. The crystal structure of compound 6 (pink sticks) bound to its receptor (silver) is compared to the predicted poses. Pose alignments were obtained by aligning the crystal receptor with 3I65 using PyMOL [22]. (a) The pharmacophore-aligned pose has a heavy-atom RMSD to the crystal pose of 1.77Å while (b) the pose minimized with a flexible ARG-265 has an RMSD of 1.18Å.

The predicted poses are compared to the actual crystal pose (PDB: 4RYH) in Fig 5. The pharmacophore-aligned pose has a heavy atom RMSD of 1.7Å, which is improved to 1.18Å by the pose minimized with a flexible ARG-165. As predicted by the pharmacophore model, compound 6 makes a hydrogen bond to HIS-185. Unsurprisingly, ARG-265 exhibits a significantly different side-chain conformation than in 3I65. The minimized residue, shown in Fig 5(b) does not recapitulate this conformation, as it seems that a smaller movement was sufficient to eliminate the steric clash with the nitro group of compound 6. Interestingly, when smina is used to dock compound 6 to its cognate receptor, the top ranked pose has an RMSD of 2.0Å. In contrast, when the pose of Fig 5(b) is further minimized against the cognate receptor, its RMSD improves to 0.93Å. This illustrates the benefit of incorporating information from known structures such as pharmacophoric constraints when performing pose prediction.

## Experimental Validation

**Fig 6:**
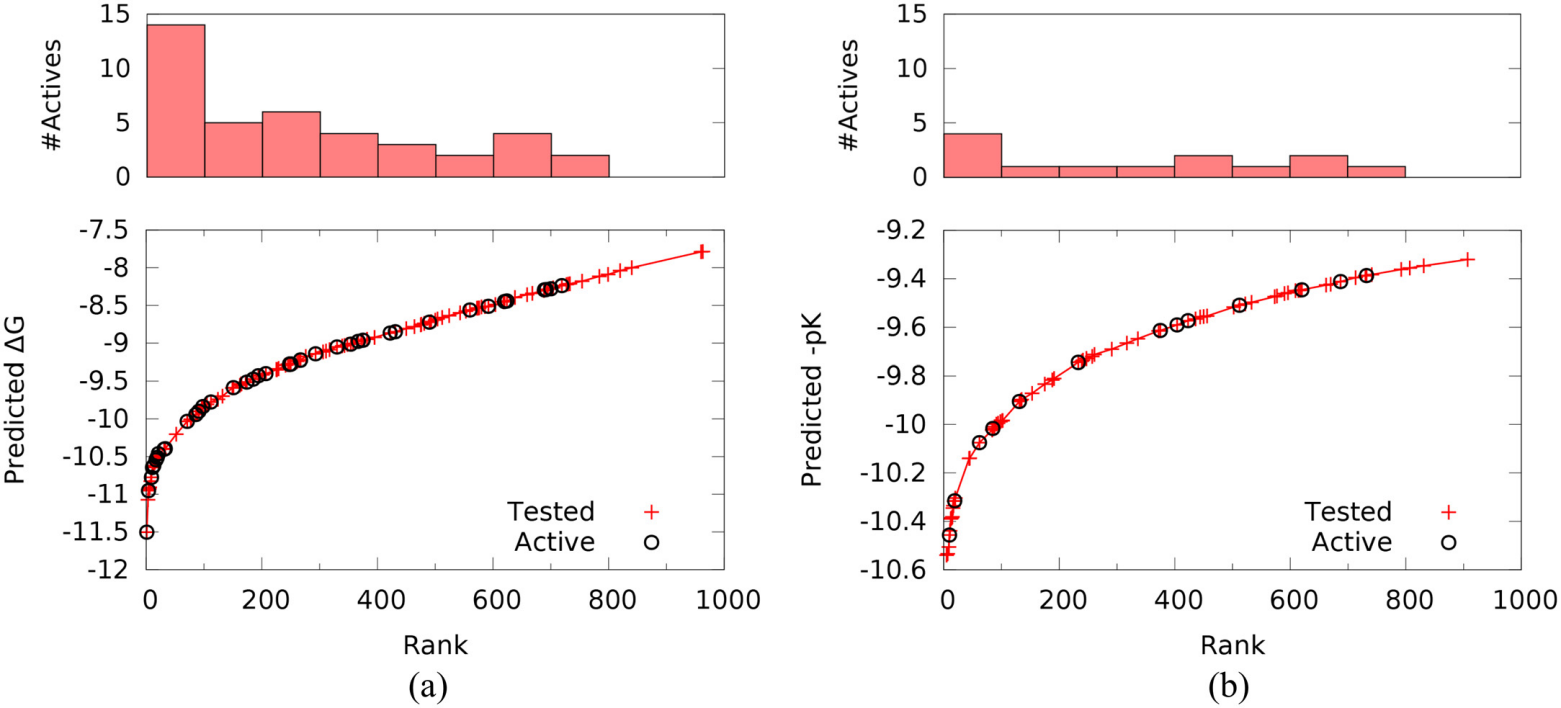
The distribution of tested and active compounds within the submitted (a) Vina ranked and (b) custom ranked compounds. For each ranking 1000 compounds were submitted from which the TDT organizers sampled a total of 167 compounds for testing. Within the Vina ranking, better scoring compounds are more likely to be active. No such enrichment is observed for the custom ranking.

The TDT organizers selected a total of 167 compounds for purchase and testing. 120 compounds were selected from the ranked list generated with Vina scoring while 72 compounds were selected from the custom scoring list. 25 selected compounds were present in both lists. The available compounds that were ranked in the top 20 in both lists were selected and then additional compounds were selected randomly from the remainder. The distribution of tested and active compounds for both rankings and their relationship to the predicted binding affinity is shown in Fig 6.

As shown in Table 3, the overall hit rate was 27%, with the compounds selected by Vina demonstrating a hit rate of 33%. Neither ranking method produced a statistically significant correlation between predicted affinity and measured affinity. However, the Vina ranking was capable of identifying an enriched subset: 15 of the 30 best ranked tested compounds were active while only 7 of the 30 lowest ranked tested compounds were active. In contrast, active compounds were essentially uniformly distributed throughout the custom ranked set.

**Table 3:** The number of compounds found to demonstrate inhibition at 10μM overall and with respect to the two different methods of ranking compounds.

	Screened	Active	Hit Rate	Novel Actives	Novel Hit Rate
**Total**	167	45	26.9%	9	5.4%
**Vina**	120	40	33.3%	5	4.2%
**Custom**	72	13	18.1%	5	6.9%
**Both**	25	8	32%	1	4%

**Fig 7:**
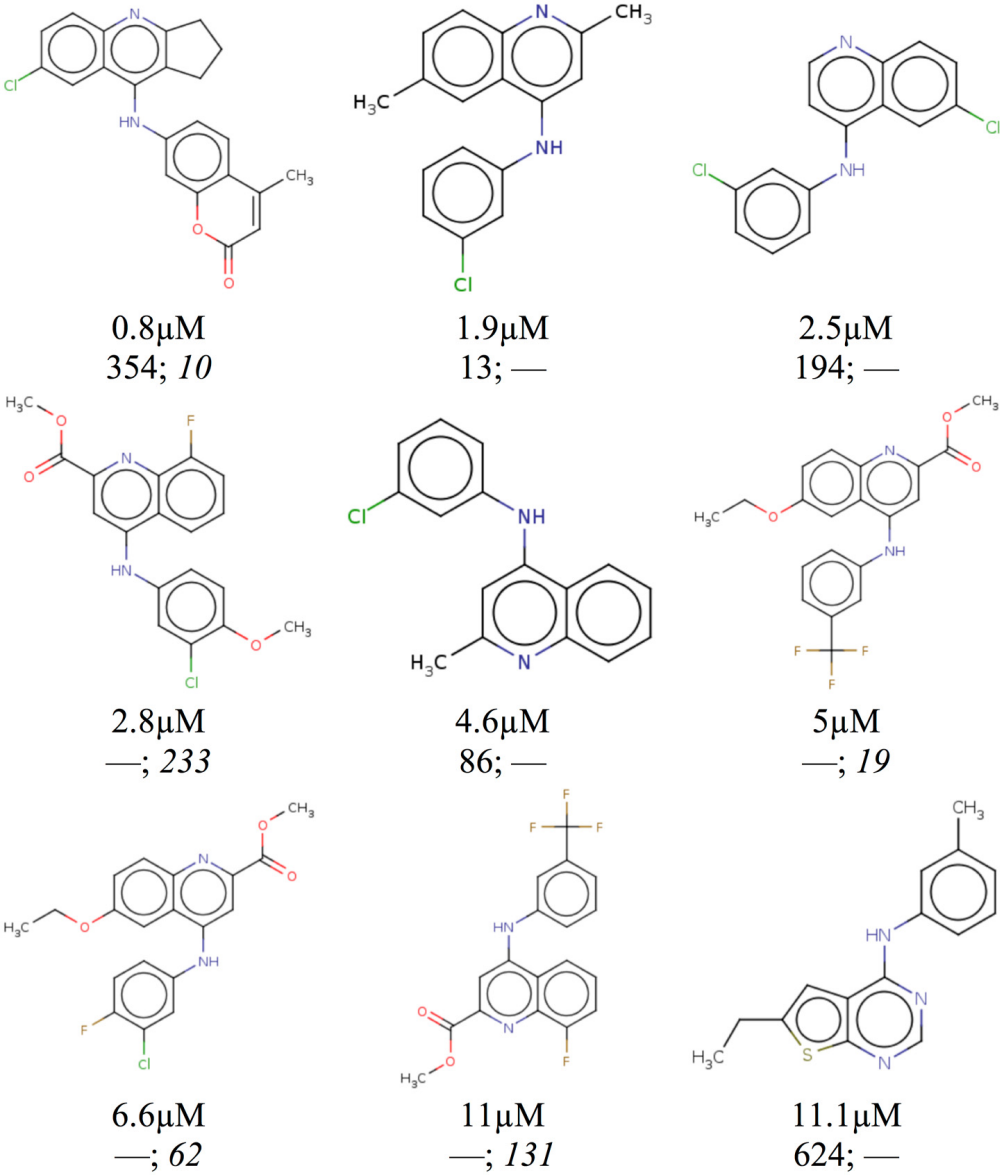
The nine active compounds with unpublished chemical scaffolds identified by the exercise. Compounds are shown with their measured IC_50_ and their rank within the Vina ranked list and the custom scored list (italic). The one compound present in both lists was the only novel compound to demonstrate sub-micromolar affinity. All compounds have a Tanimoto similarity coefficient of less than 0.16 with respect to the ligand used to define the pharmacophore as computed by OpenBabel with FP2 fingerprints.

Of the 45 identified active compounds, 9 differed significantly from previously published chemical scaffolds. These novel compounds are shown in Fig 7. Interestingly, the most potent novel compound was the only compound of these 9 that was selected by both the Vina ranking and the custom ranking. The difference in hit rates between the two rankings for novel compounds, as shown in Table 3, was less pronounced than the overall hit rate. This may be partially due to the fact than the Vina ranking selected twice as many triazolopyrimidine containing compounds. In fact, the compound top ranked by Vina scoring was the same triazolopyrimidine compound that was used to build the pharmacophore model and the co-crystal of which was used for minimization. This suggests that the Vina scoring function, which was largely parameterized on redocking performance, may have a bias towards compounds with high structural similarity to the cognate ligand of the receptor used for minimization. Given the propensity of similar compounds to have similar activities, such a similarity bias is likely to improve hit rates. However, this bias may also prove counter-productive in the search for novel compounds, as indicated by the differing hit rates for novel compounds shown in Table 3.

## Discussion

The 2012 TDT competition provided a unique opportunity to evaluate the effectiveness of interactive drug discovery within an educational setting. With limited experience, undergraduate and high school students were able to generate informative pharmacophores with high retrieval rates on a benchmark database through iterative experimentation. The best pharmacophore identified in this exercise was incorporated in a virtual screening exercise that ultimately achieved an overall hit rate of 27%. It was clear from the classroom interactions that the students would not have been able to identify informative pharmacophores without the real-time, interactive feedback they received from the online pharmacophore screening tools. We speculate that expert users would stand to gain even more from such tools as they have the experience and understanding to optimize for features beyond a simple F1 score.

The TDT competition provided a unique opportunity to participate in a truly blinded, prospective evaluation of virtual screening. We took advantage of this opportunity by submitting two rankings of compounds. Compounds were selected and ranked purely on their minimized score with respect to one of two scoring functions after being aligned to the pharmacophore. Since no other criteria were used in selecting compounds, the results provide an unbiased view of the efficacy of the two scoring functions at selecting active compounds. Interestingly, there was no clearly superior scoring function. Although the Vina scoring function achieved the highest hit rate and compounds that scored better with Vina were more likely to be active, the Vina scoring function wasn't as successful at identifying novel compounds that were structurally distinct from the reference structure used for pharmacophore modeling and minimization. This underscores that the development of effective and predictive scoring functions remains a challenging task [23].

Despite the challenges, the independent and blind validation of our predictions provides strong support for the robustness of our approach. Moreover, our submission succeeded in its dual goals of drug discovery and education. Several novel inhibitors of DHODH were identified. Interactive pharmacophore modeling proved a powerful, engaging and instructive exercise. Full tutorial materials are available from the TDT website (http://www.tdtproject.org).

## References

[1] Gero AM, O'Sullivan WJ. Purines and pyrimidines in malarial parasites. Blood cells. 1990;16(2–3):467–84; discussion 85–98. Epub 1990/01/01. . 2257323

[2] Coteron JM, Marco M, Esquivias J, Deng X, White KL, White J, et al Structure-guided lead optimization of triazolopyrimidine-ring substituents identifies potent Plasmodium falciparum dihydroorotate dehydrogenase inhibitors with clinical candidate potential. J Med Chem. 2011;54(15):5540–61. Epub 2011/06/24. 10.1021/jm200592f 21696174 PMC3156099

[3] Booker ML, Bastos CM, Kramer ML, Barker RH Jr., Skerlj R, Sidhu AB, et al Novel inhibitors of Plasmodium falciparum dihydroorotate dehydrogenase with anti-malarial activity in the mouse model. J Biol Chem. 2010;285(43):33054–64. Epub 2010/08/13. 10.1074/jbc.M110.162081 20702404 PMC2963363

[4] Deng X, Gujjar R, El Mazouni F, Kaminsky W, Malmquist NA, Goldsmith EJ, et al Structural plasticity of malaria dihydroorotate dehydrogenase allows selective binding of diverse chemical scaffolds. J Biol Chem. 2009;284(39):26999–7009. Epub 2009/07/31. 10.1074/jbc.M109.028589 19640844 PMC2785385

[5] Phillips MA, Gujjar R, Malmquist NA, White J, El Mazouni F, Baldwin J, et al Triazolopyrimidine-based dihydroorotate dehydrogenase inhibitors with potent and selective activity against the malaria parasite Plasmodium falciparum. J Med Chem. 2008;51(12):3649–53. Epub 2008/06/05. 10.1021/jm8001026 18522386 PMC2624570

[6] Gujjar R, Marwaha A, El Mazouni F, White J, White KL, Creason S, et al Identification of a metabolically stable triazolopyrimidine-based dihydroorotate dehydrogenase inhibitor with antimalarial activity in mice. J Med Chem. 2009;52(7):1864–72. Epub 2009/03/20. 10.1021/jm801343r 19296651 PMC2746568

[7] Gujjar R, El Mazouni F, White KL, White J, Creason S, Shackleford DM, et al Lead optimization of aryl and aralkyl amine-based triazolopyrimidine inhibitors of Plasmodium falciparum dihydroorotate dehydrogenase with antimalarial activity in mice. J Med Chem. 2011;54(11):3935–49. Epub 2011/04/27. 10.1021/jm200265b 21517059 PMC3124361

[8] Baldwin J, Michnoff CH, Malmquist NA, White J, Roth MG, Rathod PK, et al High-throughput Screening for Potent and Selective Inhibitors of Plasmodium falciparum Dihydroorotate Dehydrogenase. Journal of Biological Chemistry. 2005;280(23):21847–53. 10.1074/jbc.M501100200 15795226

[9] Koes DR, Camacho CJ. ZINCPharmer: pharmacophore search of the ZINC database. Nucleic acids research. 2012;40(Web Server issue):W409–14. Epub 2012/05/04. 10.1093/nar/gks378 22553363 PMC3394271

[10] Irwin JJ, Sterling T, Mysinger MM, Bolstad ES, Coleman RG. ZINC: a free tool to discover chemistry for biology. J Chem Inf Model. 2012;52(7):1757–68. Epub 2012/05/17. 10.1021/ci3001277 22587354 PMC3402020

[11] Norman TC, Bountra C, Edwards AM, Yamamoto KR, Friend SH. Leveraging crowdsourcing to facilitate the discovery of new medicines. Sci Transl Med. 2011;3(88):88mr1 10.1126/scitranslmed.3002678 . 21697527

[12] Haigh JA, Pickup BT, Grant JA, Nicholls A. Small molecule shape-fingerprints. J Chem Inf Model. 2005;45(3):673–84. Epub 2005/06/01. 10.1021/ci049651v . 15921457

[13] Shim J, Mackerell AD Jr. Computational ligand-based rational design: Role of conformational sampling and force fields in model development. Medchemcomm. 2011;2(5):356–70. 10.1039/C1MD00044F 21716805 PMC3123535

[14] Sheridan RP, Kearsley SK. Why do we need so many chemical similarity search methods? Drug discovery today. 2002;7(17):903–11. 12546933 10.1016/s1359-6446(02)02411-x

[15] Koes DR, Camacho CJ. Pharmer: Efficient and Exact Pharmacophore Search. Journal of Chemical Information and Modeling. 2011:null–null. 10.1021/ci200097m PMC3124593 21604800

[16] Koes DR, Baumgartner MP, Camacho CJ. Lessons learned in empirical scoring with smina from the CSAR 2011 benchmarking exercise. J Chem Inf Model. 2013;53(8):1893–904. Epub 2013/02/06. 10.1021/ci300604z 23379370 PMC3726561

[17] Trott O, Olson AJ. AutoDock Vina: improving the speed and accuracy of docking with a new scoring function, efficient optimization, and multithreading. J Comput Chem. 2010;31(2):455–61. Epub 2009/06/06. 10.1002/jcc.21334 19499576 PMC3041641

[18] Dunbar JB Jr., Smith RD, Yang CY, Ung PM, Lexa KW, Khazanov NA, et al CSAR benchmark exercise of 2010: selection of the protein-ligand complexes. J Chem Inf Model. 2011;51(9):2036–46. Epub 2011/07/07. 10.1021/ci200082t 21728306 PMC3180202

[19] O'Boyle NM, Banck M, James CA, Morley C, Vandermeersch T, Hutchison GR. Open Babel: An open chemical toolbox. J Cheminform. 2011;3:33 Epub 2011/10/11. 10.1186/1758-2946-3-33 21982300 PMC3198950

[20] Deng X, Kokkonda S, El Mazouni F, White J, Burrows JN, Kaminsky W, et al Fluorine modulates species selectivity in the triazolopyrimidine class of Plasmodium falciparum dihydroorotate dehydrogenase inhibitors. Journal of medicinal chemistry. 2014;57(12):5381–94. 10.1021/jm500481t 24801997 PMC4079327

[21] Baldwin J, Michnoff CH, Malmquist NA, White J, Roth MG, Rathod PK, et al High-throughput screening for potent and selective inhibitors of Plasmodium falciparum dihydroorotate dehydrogenase. The Journal of biological chemistry. 2005;280(23):21847–53. 10.1074/jbc.M501100200 . 15795226

[22] Schrödinger L. The PyMOL Molecular Graphics System, Version 1.6. 2014.

[23] Smith RD, Dunbar JB Jr., Ung PM, Esposito EX, Yang CY, Wang S, et al CSAR benchmark exercise of 2010: combined evaluation across all submitted scoring functions. J Chem Inf Model. 2011;51(9):2115–31. Epub 2011/08/04. 10.1021/ci200269q 21809884 PMC3186041

